# Low temperature plasma irradiation products of sodium lactate solution that induce cell death on U251SP glioblastoma cells were identified

**DOI:** 10.1038/s41598-021-98020-w

**Published:** 2021-09-16

**Authors:** Hiromasa Tanaka, Yugo Hosoi, Kenji Ishikawa, Jun Yoshitake, Takahiro Shibata, Koji Uchida, Hiroshi Hashizume, Masaaki Mizuno, Yasumasa Okazaki, Shinya Toyokuni, Kae Nakamura, Hiroaki Kajiyama, Fumitaka Kikkawa, Masaru Hori

**Affiliations:** 1 Center for Low-Temperature Plasma Sciences, Nagoya University, Furo-cho, Chikusa-ku, Nagoya, 464-8601 Japan; 2 Graduate School of Engineering, Nagoya University, Furo-cho, Chikusa-ku, Nagoya, 464-8603 Japan; 3 Institute of Nano-Life-Systems, Institute of Innovation for Future Society, Nagoya University, Nagoya, 464-8601 Japan; 4 Graduate School of Bioagricultural Sciences, Nagoya University, Furo-cho, Chikusa-ku, Nagoya, 464-8603 Japan; 5 Department of Applied Biological Chemistry, Graduate School of Agricultural and Life Sciences, The University of Tokyo, 1-1-1 Yayoi, Bunkyo-ku, Tokyo, 113-8657 Japan; 6 Institutes of Innovation for Future Society, Nagoya University, Furo-cho, Chikusa-ku, Nagoya, 464-8601 Japan; 7 Center for Advanced Medicine and Clinical Research, Nagoya University Hospital, 65 Tsurumai-cho, Showa-ku, Nagoya, 466-8550 Japan; 8 Department of Pathology and Biological Responses, Nagoya University Graduate School of Medicine, 65 Tsurumai-cho, Showa-ku, Nagoya, 466-8550 Japan; 9 Department of Obstetrics and Gynecology, Nagoya University Graduate School of Medicine, 65 Tsurumai-cho, Showa-ku, Nagoya, 466-8550 Japan

**Keywords:** Biochemistry, Cancer, Cell biology, Chemical biology, Engineering

## Abstract

Low-temperature plasma is being widely used in the various fields of life science, such as medicine and agriculture. Plasma-activated solutions have been proposed as potential cancer therapeutic reagents. We previously reported that plasma-activated Ringer’s lactate solution exhibited selective cancer-killing effects, and that the plasma-treated L-sodium lactate in the solution was an anti-tumor factor; however, the components that are generated through the interactions between plasma and L-sodium lactate and the components responsible for the selective killing of cancer cells remain unidentified. In this study, we quantified several major chemical products, such as pyruvate, formate, and acetate, in plasma-activated L-sodium lactate solution by nuclear magnetic resonance analysis. We further identified novel chemical products, such as glyoxylate and 2,3-dimethyltartrate, in the solution by direct infusion-electrospray ionization with tandem mass spectrometry analysis. We found that 2,3-dimethyltartrate exhibited cytotoxic effects in glioblastoma cells, but not in normal astrocytes. These findings shed light on the identities of the components that are responsible for the selective cytotoxic effect of plasma-activated solutions on cancer cells, and provide useful data for the potential development of cancer treatments using plasma-activated L-sodium lactate solution.

## Introduction

Low-temperature plasma (LTP) is being widely used in various fields, such as medicine and agriculture, which are now referred to as plasma medicine and plasma agriculture, respectively^1–5^. LTP acts on biological surfaces directly or indirectly through solutions^6–9^. We have previously reported that plasma-activated medium (PAM) exhibited anti-tumor effects on cancer cells^10–16^, and have developed plasma-activated Ringer’s lactate solution (PAL) for clinical applications^17–20^. Many in vitro and in vivo studies have demonstrated that both PAM and PAL exhibited anti-tumor effects. We recently reported that PAM induced macrophage infiltration in disseminated lesions in our mouse model of intraperitoneally disseminated ovarian cancer, and proposed an intraperitoneal washing therapy using PAL for the treatment of ovarian cancer^20^. We have also reported that PAL induced ferroptosis in malignant mesothelioma cells via lipid peroxidation mediated by lysosomal nitric oxide-derived oxidants^21^. Although both PAM and PAL exhibited cytotoxic effects, the mechanisms of cell death appear to be different. For example, PAM induced oxidative stress-dependent cell death, while PAL induced oxidative stress-independent cell death in glioblastoma cells^19^. Comprehensive understanding of the mechanisms is essential for the clinical application of plasma-activated solutions as cancer treatments.

**Figure 1:**
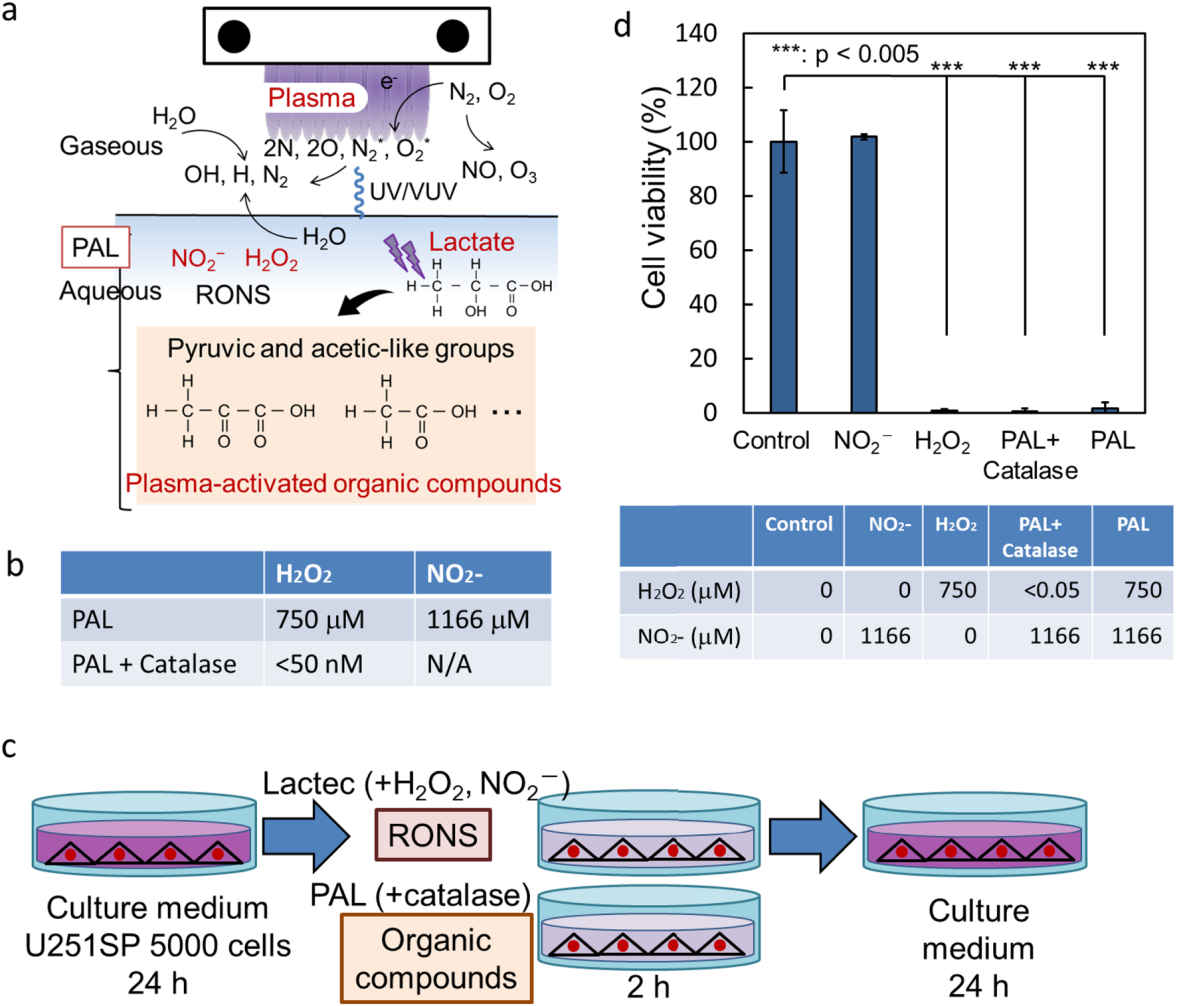
The effect of RONS in PAL. (**a**) Schematic of the chemical components in Plasma-activated Ringer’s lactate solution (PAL). (**b**) Concentrations of hydrogen peroxide and nitrite in PAL with and without catalase. (**c**) Experimental procedure to measure the cytotoxicity of Reactive oxygen and nitrogen species (RONS) and PAL with and without catalase. (**d**) Cytotoxic effects of RONS in PAL. Lactec: Ringer’s lactate solution.

Plasma–liquid interactions have been extensively studied^22–24^. Since air contains nitrogen, oxygen, and water, short-lived radicals, such as OH and NO, and long-lived reactive species, which contain N, O, and H (reactive oxygen and nitrogen species (RONS)), are generated in the gaseous phase^25^, and the main products from plasma–liquid interactions in plasma-activated solutions are hydrogen peroxide (H_2_O_2_), nitrite (NO_2_^−^), and nitrate (NO_3_^−^; Fig. 1a). We previously reported that hydrogen peroxide in PAM was partially responsible for the cytotoxicity, while nitrite was not responsible for the cytotoxicity at all; however, a synergetic effect between hydrogen peroxide and nitrite was observed. In addition, other unknown components in PAM are also responsible for the cytotoxicity^26^. Culture medium contains about 30 components, including inorganic compounds, vitamins, and amino acids. Interactions between LTP and amino acids have been extensively studied. However, so far, most of the studies have examined the loss of function of amino acids as nutrients. For example, methionine in medium was oxidized by LTP treatment^27^. Ringer’s lactate solution contains sodium chloride, potassium chloride, calcium chloride, and L-sodium lactate, and we have previously reported that the reaction products between plasma and L-sodium lactate were anti-tumor factors in PAL^17^. We have also reported that the concentration of hydrogen peroxide in PAL depends on the concentration of L-sodium lactate in irradiated Ringer’s lactate solution^28^. L-sodium lactate is an organic chemical, and the plasma treatment of L-sodium lactate is thought to cause chemical bonds to dissociate and associate; however, it remains a big mystery what components are generated by the interactions between plasma and L-sodium lactate, and what components are responsible for the selective killing of cancer cells by PAL.

In this study, we performed a nuclear magnetic resonance (NMR) analysis and mass spectrometry analysis to identify the components in PAL. We identified glyoxylate and 2,3-dimethyltartrate, in addition to pyruvate, acetate, and formate. 2,3-Dimethyltartrate exhibited selective killing of cancer cells, while glyoxylate killed both glioblastoma cells and normal astrocytes. These results suggest that 2,3-dimethyltartrate is a key factor that is responsible for the selective killing of cancer cells by PAL.

## Results

### The cytotoxic effect of the RONS in PAL on glioblastoma cells

We measured the concentrations of hydrogen peroxide and nitrite in PAL. We found that the concentration of hydrogen peroxide in the PAL was 750 μM and the concentration of nitrite in the PAL was 1166 μM (Fig. 1b). To eliminate the cytotoxic effect of hydrogen peroxide in PAL, we treated PAL with catalase (0.5 mg/ml in PAL) for 2 h before glioblastoma cells were treated with the PAL, and the hydrogen peroxide concentration was less than 50 nM after catalase treatment. We assessed the cytotoxic effect of each RONS in PAL by the MTS assay (Fig. 1c). We found that 1166 μM nitrite did not exhibit any cytotoxicity, while 750 μM hydrogen peroxide as well as PAL with catalase (hydrogen peroxide concentration of < 50 nM) completely killed the glioblastoma cells (Fig. 1d). These results suggested that components other than RONS in PAL are responsible for the cytotoxicity of PAL.

### NMR analysis of the components of plasma-treated L-sodium lactate solution

**Figure 2:**
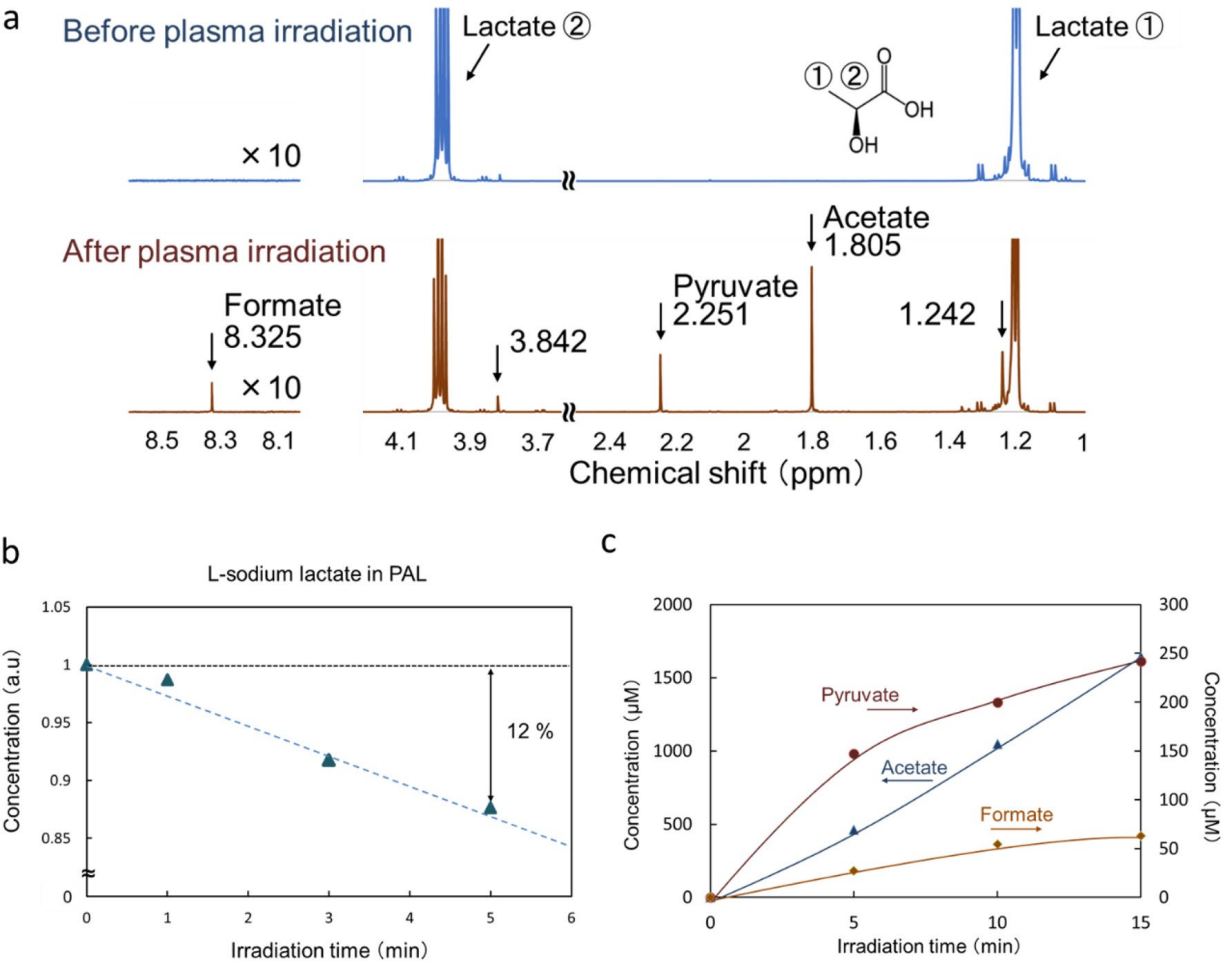
NMR analysis of PAL. (**a**) Chemical shift before and after plasma irradiation when L-sodium lactate was treated with LTP for 5 min. (**b**) The consumption rate of L-sodium lactate increases with increasing irradiation time of the plasma treatment. (**c**) The concentrations of pyruvate, acetate, and formate increase with increasing irradiation time of the plasma treatment.

We previously reported that plasma-treated L-sodium lactate contained more acetyl (CH_3_CO) and pyruvic acid-like groups (CH_3_COCOOH) than untreated L-sodium lactate solution based on NMR analyses^17^. To investigate quantitative aspects of plasma-treated products, we performed ^1^H NMR analyses with different plasma irradiation times (Fig. 2). Figure 2b shows the differences in the ^1^H NMR peak patterns before and after plasma irradiation of L-sodium lactate solution (L-sodium lactate in deuterium oxide). Signals from formate, pyruvate, and acetate clearly increased after plasma irradiation. The peak area of signals from L-sodium lactate, which is proportional to the concentration of L-sodium lactate, was measured for different plasma irradiation times (Fig. 2c). When L-sodium lactate was irradiated with plasma for 5 min, 12% of the L-sodium lactate was consumed. Figure 2c shows the concentrations of formate, pyruvate, and acetate in plasma-treated L-sodium lactate solution with different irradiation times; after 15 min of plasma irradiation, the concentrations were 60 μM for formate, 250 μM for pyruvate, and 1500 μM for acetate.

### Determination of the components of PAL using direct infusion-electrospray ionization (ESI) with tandem mass spectrometry (MS/MS)

**Figure 3:**
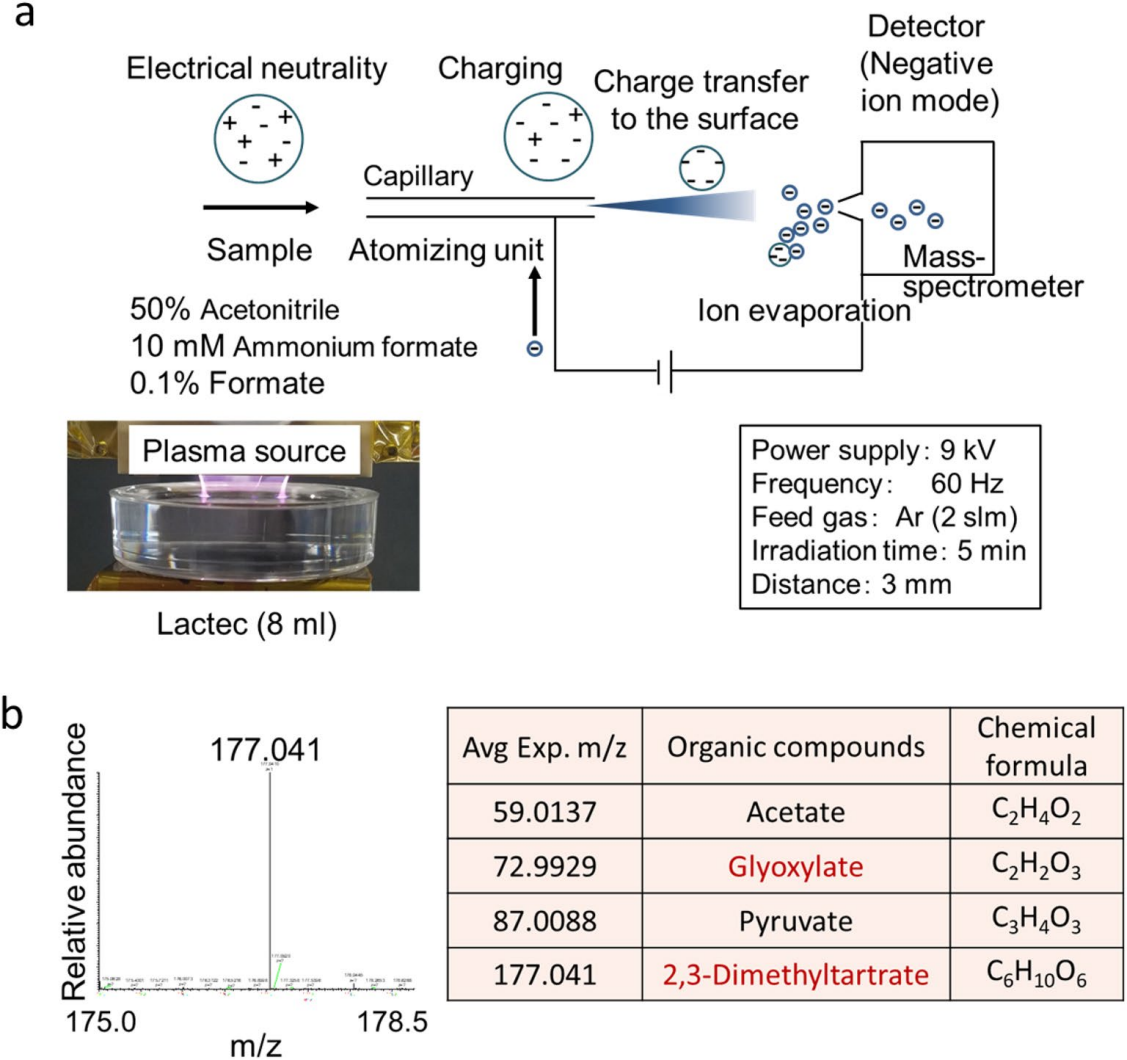
Direct infusion-ESI–MS/MS analysis of PAL. (**a**) Schematic of the direct infusion-ESI–MS/MS analysis of PAL. (**b**) Acetate (*m/z* 59.0137), glyoxylate (*m/z* 72.9929), pyruvate (*m/z* 87.0088), and 2,3-dimethyltartrate (*m/z* 177.041) were identified in PAL. Lactec: Ringer’s lactate solution.

To determine the components of PAL, we performed direct infusion-ESI–MS/MS analysis (Fig. 3). We used 50% acetonitrile, 10 mM ammonium formate, and 0.1% formate for dissolution. As expected from the NMR results, acetate (*m/z* 59.0137) and pyruvate (*m/z* 87.0088) were detected in PAL (Figure S1 and Fig. 3b). In addition, glyoxylate (*m/z* 72.9929) and 2,3-dimethyltartrate (*m/z* 177.041) were also detected in PAL. Due to the detection limit of the MS/MS system (*m/z* < 50), formate was not detected in this experiment.

### Anti-tumor effects of the products of plasma-treated L-sodium lactate

**Figure 4:**
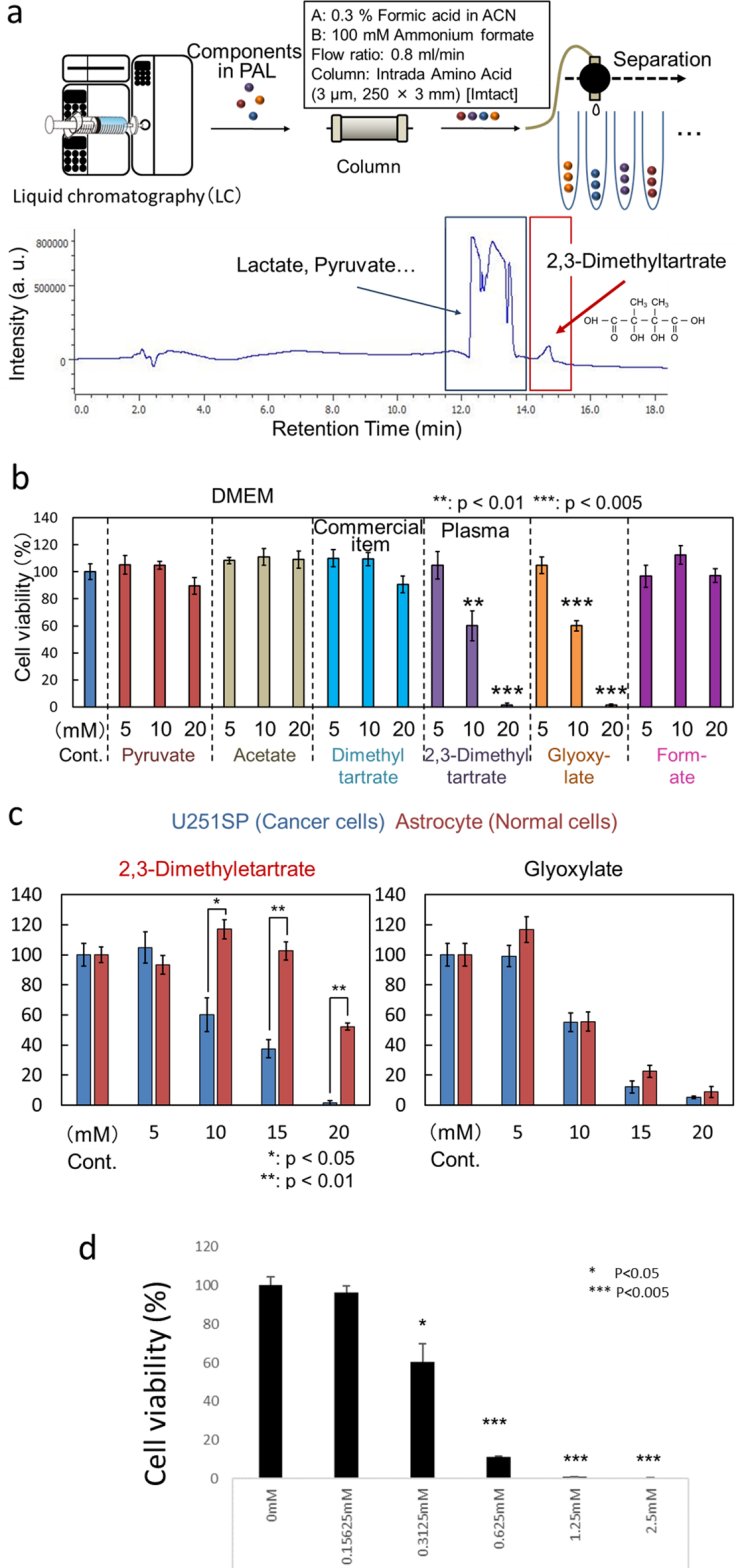
Anti-tumor effect of each component of PAL. (**a**) Schematic of the purification procedure for 2,3-dimetyltartrate in plasma-activated L-sodium lactate solution. (**b**) The cell viability of U251SP glioblastoma cells treated with 5, 10, or 20 mM of pyruvate, acetate, dimethyltartrate, 2,3-dimethyltartrate, glyoxylate, or formate in culture medium. (**c**) Cell viability of U251SP glioblastoma cells and normal astrocytes treated with 5, 10, 15, or 20 mM of 2,3-dimethyltartrate or glyoxylate in culture medium. (**d**) The cell viability of U251SP glioblastoma cells treated with 0.15625, 0.3125, 0.625, 1.25, or 2.5 mM of synthesized 2,3-dimethyltartrate in Lactec.

To investigate which products in PAL are responsible for the anti-tumor effects, we performed a cell viability assay (Fig. 4). 2,3-Dimethyltartrate is not commercially available, so we instead purchased the commercially available dimethyltartrate, in addition to the commercially available pyruvate, acetate, and formate, and used them for the cell viability assay. In addition, we purified 2,3-dimethyltartrate from plasma-activated L-sodium lactate solution by high-performance liquid chromatography (HPLC; Fig. 4a and Figure S2). L-sodium lactate was detected in the 12-to-13-min and 13-to-14-min fractions. 2,3-Dimethyltartrate was detected in the 13-to-14-min and 14-to-15-min fractions, so we purified 2,3-dimethyltartrate from the 14-to-15 min fraction (Figure S2).

Since L-sodium lactate is present in Ringer’s lactate solution (Lactec) at a concentration of 28 mM, we evaluated the cytotoxic effect of the purchased dimethyltartrate, pyruvate, acetate, and formate, and the purified 2,3-dimethyltartrate at the concentrations of 5, 10, and 20 mM on U251SP glioblastoma cells using the MTS assay (Fig. 4b). We found that pyruvate, acetate, formate, and dimethyltartrate had no cytotoxic effect on U251SP glioblastoma cells, while glyoxylate and 2,3-dimethyltartrate exhibited a cytotoxic effect on the cells. To investigate the tumor specificity of the cytotoxic effects of the compounds, we treated normal human astrocytes as well as U251SP glioblastoma cells with 5, 10, 15, and 20 mM of glyoxylate or 2,3-dimethyltartrate (Fig. 4c). Interestingly, 2,3-dimethyltartrate exhibited much higher cytotoxicity in U251SP glioblastoma cells than in normal astrocytes, while glyoxylate exhibited almost the same level of cytotoxicity in both U251SP glioblastoma cells and normal astrocytes. We further synthesized 2,3-dimethyltartrate by organic synthesis. We treated U251SP glioblastoma cells with 0.15625, 0.3125, 0.625, 1.25 or 2.5 mM of synthesized 2,3-dimethyltartrate in Lactec (Fig. 4d). Interestingly, low concentration of 2,3-dimethyltartrate in Lactec (> 0.3125 mM) was enough to exhibit anti-tumor effect on U251SP glioblastoma cells while relatively high concentration of 2,3-dimethyltartrate in culture medium (> 10 mM) was needed (Fig. 4c). These results suggested that 2,3-dimethyltartrate in PAL is responsible for the selective killing of cancer cells by PAL.

**Figure 5:**
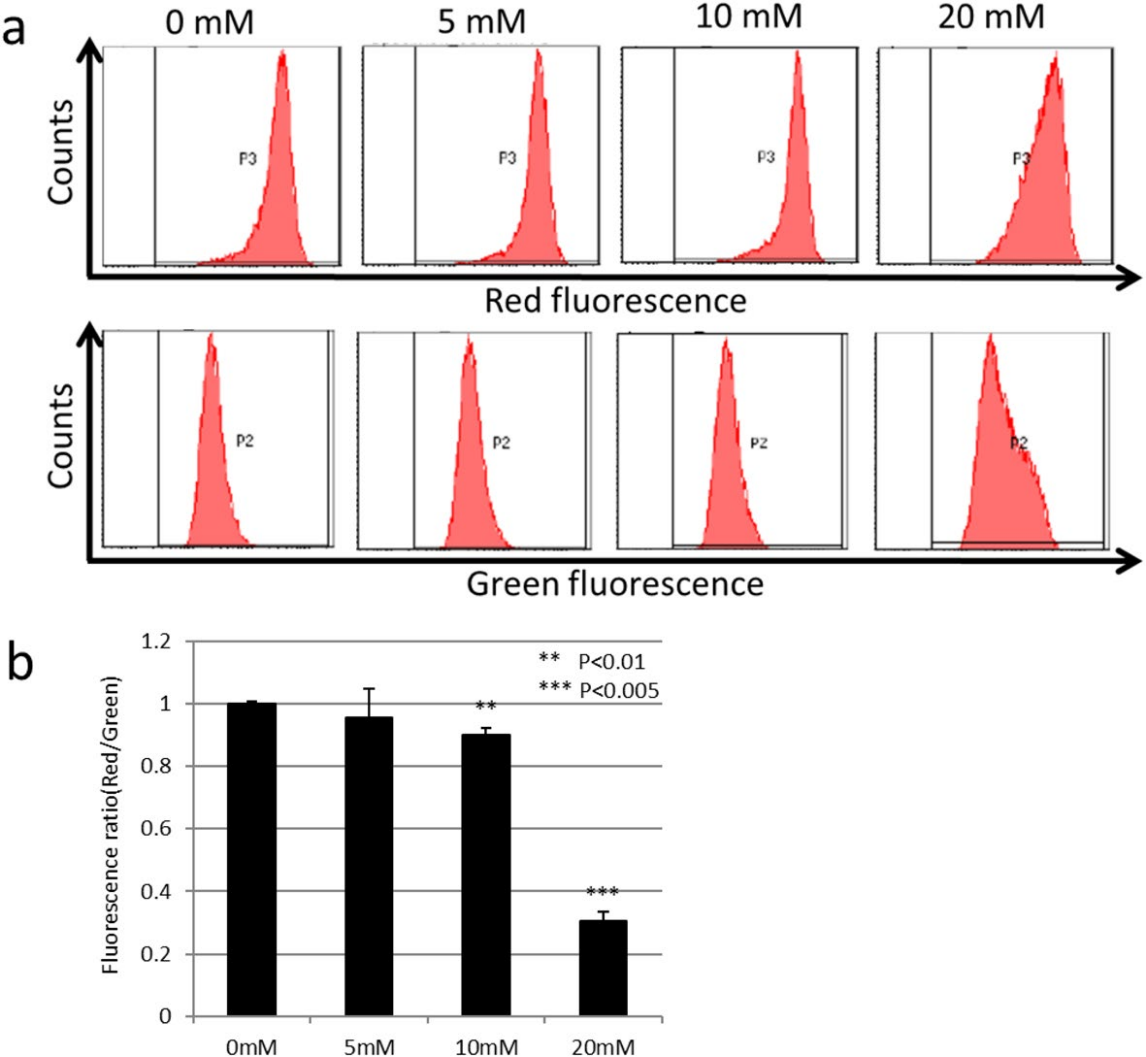
Mitochondrial injury induced by synthesized 2,3-dimethyltartrate. (**a**) Mitochondrial injury induced by 5, 10, or 20 mM of synthesized 2,3-dimethyltartrate. U251SP glioblastoma cells were stained with the JC-1 probe, and red and green fluorescence were measured by the flow cytometry. (**b**) Fluorescence intensities were measured, and red/green fluorescence ratios were calculated. Values are presented as mean ± SD (n = 3).

To elucidate the mechanisms of cell death by 2,3-dimethyltartrate, we investigate the mitochondrial membrane potential using the JC-1 probe (Fig. 5). We treated U251SP glioblastoma cells with 5, 10, or 20 mM of the synthesized 2,3-dimethyltartrate in culture medium, and we stained with the fluorescence dye JC-1 and measured the fluorescence of red and green using the flow cytometry (Fig. 5a). Fluorescence ratio (Red/Green) decreased in 20 mM of 2,3-dimethyltartrate-treated U251SP cells (Fig. 5b). These results suggest that mitochondrial injury was induced by 2,3-dimethyltartrate.

## Discussion

**Figure 6:**
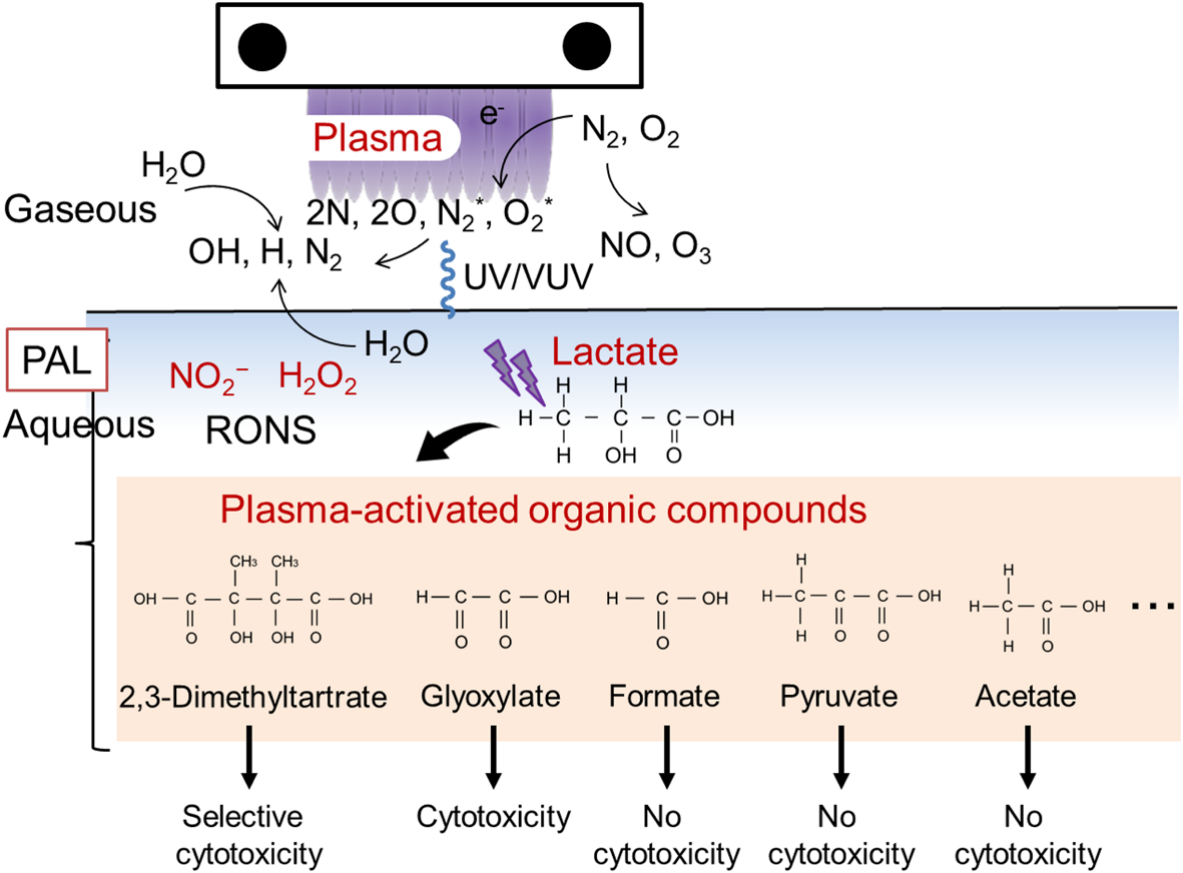
Components in PAL identified by the NMR analysis and direct infusion-ESI–MS/MS analysis. Among the components of PAL, 2,3-dimethyltartrate exhibited selective cytotoxicity on cancer cells, glyoxylate exhibited cytotoxicity on both cancer and normal cells, and formate, pyruvate, and acetate did not exhibit any cytotoxicity on cells.

In this study, we characterized several organic chemicals that are produced from the plasma treatment of L-sodium lactate solution or Ringer’s lactate solution. Figure 6 summarizes the products in PAL. NMR and direct infusion-ESI–MS analyses revealed that acetate, pyruvate, and formate were produced through the reactions between LTP and L-sodium lactate (Figs. 2 and 3); however, these products did not exhibit any cytotoxicity on glioblastoma cells even at 20 mM (Fig. 4b). We estimated that the concentration of L-sodium lactate decreased by around 3 mM in Ringer’s lactate solution, which normally contains 28 mM L-sodium lactate, while the concentrations of acetate, pyruvate, and formate increased by about 500 μM, 150 μM, and 30 μM, respectively, after LTP treatment for 5 min (Fig. 2). In addition, direct infusion-ESI–MS/MS revealed that new products, namely, glyoxylate and 2,3-dimethyltartrate, were produced. When we treated Lactec with plasma for 5 min, 12% of L-sodium lactate was consumed (Fig. 2b), so we can estimate that the concentration of 2,3-dimethyltartrate in the PAL was less than 1.68 mM and the concentration of glyoxylate in the PAL was less than 3.36 mM. When we treated Lactec with plasma for 3 min, 9% of L-sodium lactate was consumed (Fig. 2b), so we can also estimate that the concentration of 2,3-dimethyltartrate in the PAL was less than 1.26 mM and the concentration of glyoxylate in the PAL was less than 2.52 mM. Glyoxylate serves as an intermediate in various metabolic pathways, although high concentrations of this metabolite are toxic to cells^29^. Indeed, glyoxylate exhibited cytotoxicity on both glioblastoma cells and normal astrocytes (Fig. 4c). To our knowledge, 2,3-dimetyltartrate has not been reported as a cytotoxic biochemical compound, and it is not commercially available for purchase. We purified 2,3-dimetyltartrate using ultra-performance liquid chromatography, and we demonstrated that the purified 2,3-dimetyltartrate in culture medium exhibited a cytotoxic effect on U251SP glioblastoma cells, but it did not exhibit a cytotoxic effect on normal astrocytes (Fig. 4c). These results suggest that 2,3-dimetyltartrate is responsible for the selective killing of cancer cells. Meanwhile, 20 mM of 2,3-dimetyltartrate in culture medium was used in these experiments, which might be higher concentration than one in PAL. We further treated U251SP glioblastoma cells with 2,3-dimetyltartrate in Lactec (Fig. 4d). Interestingly, more than 0.3125 mM of 2,3-dimetyltartrate in Lactec exhibited anti-tumor effects on the U251SP glioblastoma cells. These results suggest that 2,3-dimethyltartrate in PAL might be responsible for anti-tumor effects by PAL. Other components in PAL might also be responsible for the selective killing of cancer cells, and further experiments are needed to comprehensively understand the mechanisms of the specific anti-tumor effects of PAL.

We have previously reported that PAM induced oxidative stress-dependent cell death on U251SP glioblastoma cells, while PAL induced oxidative stress-independent cell death on them^19^. In this study, we found that 2,3-dimethyltartrate which is a product of plasma-treated L-sodium lactate is a candidate of the anti-tumor factor in PAL. However, L-sodium lactate is not the component of culture medium. Other components in culture medium should be responsible for anti-tumor effects by PAM. In the future, such anti-tumor factors in PAL and PAM should be identified and some of them might be used for clinical applications. Towards such clinical applications, further experiments such as in vivo experiments and other types of cancer cells are needed.

## Conclusion

LTP treatment causes the formation of organic chemicals derived from L-sodium lactate in PAL. We characterized several components in PAL, and found that pyruvate, acetate, and formate did not exhibit cytotoxicity against glioblastoma cells, glyoxylate exhibited cytotoxicity against both glioblastoma cells and normal astrocytes, and 2,3-dimethyltartrate exhibited cytotoxicity against glioblastoma cells, but not against normal astrocytes. These findings represent a major step towards unraveling the great mystery of how PAL causes the selective killing of cancer cells.

## Materials and methods

### Cell lines and cultures

U251SP glioblastoma cells were obtained from the Memorial Sloan-Kettering Cancer Institute (New York, NY), and were grown in Dulbecco’s Modified Eagle Medium (Sigma-Aldrich, St. Louis, MO)^30^ supplemented with 10% fetal bovine serum and penicillin (100 U/ml)-streptomycin (100 μg/ml) in an atmosphere of 5% CO_2_ at 37 °C. Human astrocytes (N7805100) were obtained from Thermo Fisher Scientific (Waltham, MA), and were grown in Gibco Astrocyte Medium in an atmosphere of 5% CO_2_ at 37 °C.

### Preparation of plasma-activated L-sodium lactate solution and PAL

Plasma-activated L-sodium lactate solution and PAL were prepared by treating the solutions with plasma as previously described^17^. The plasma source was connected to 9 kV from a 60-Hz AC high-voltage power source with argon gas flowing at a rate of 2 standard liters per min. The distance between the plasma source and the samples was fixed at L = 3 mm.

### Concentrations of hydrogen peroxide

The concentration of hydrogen peroxide was measured using the Amplex Red hydrogen peroxide/peroxidase assay kit (Thermo Fisher Scientific), and the peak fluorescence emission (excitation: 530 nm, emission: 560 nm) was monitored with a microplate reader (POWERSCAN HT, DS Pharma Biomedical, Kirkland, WA) as previously described^26^.

### Concentrations of nitrite

The concentration of nitrite was measured using the OxiSelect In Vitro Nitric Oxide (Nitrite/Nitrate) Assay Kit (Cell Biolabs Inc., San Diego, CA), and the peak fluorescence emission (excitation: 480 nm, emission: 540 nm) was monitored with a microplate reader (POWERSCAN HT, DS Pharma Biomedical) as previously described^26^.

### Cell viability assay

The effect of PAL on cell viability was measured using the Aqueous One Solution Cell Proliferation Assay kit (Promega, Madison, WI) according to the manufacturer’s instructions. Absorbance was measured at 490 nm with a microplate reader (POWERSCAN HT; DS Pharma Biomedical). For the assay, 5000 cells in 200 μl of culture medium were seeded into the wells of a 96-well plate. After 24 h of incubation, cells were treated with untreated Ringer’s lactate solution, 1166 μM nitrite in Ringer’s lactate solution, 750 μM hydrogen peroxide in Ringer’s lactate solution, or PAL with or without catalase for 2 h. Then, the culture medium was replaced with them**.** After 24 h of incubation, the cell viability was measured by the MTS assay.

### NMR analysis of plasma-activated L-sodium lactate solution

In a 60-mm dish, 28 mM L-sodium lactate in 8 ml D_2_O was treated with plasma (L = 3 mm, 2.0 slm). The ^1^H NMR spectra of untreated L-sodium lactate solution and plasma-activated L-sodium lactate solution were measured with a JNM-ECA600 spectrometer (JEOL RESONANCE, Tokyo, Japan) operated at 600 MHz for ^1^H nuclei. The integral (area under the curve) of each product peak, which is proportional to the concentration of each product, was calculated and used to quantify the concentrations of the products.

### Direct infusion-ESI–MS/ES analysis of PAL

Direct infusion-ESI–MS/MS analysis was performed with an Orbitrap Fusion mass spectrometer (Thermo Fisher Scientific) in the negative ion mode (Fig. 3a). In a 60-mm dish, 8 ml of Ringer’s lactate solution was treated with plasma (L = 3 mm, 2.0 slm) for 5 min. The sample was injected at a flow rate of 1 μl/min, and 50% acetonitrile, 10 mM ammonium formate, and 0.1% formate was used for sample dissolution.

### Purification of 2,3-dimethyltartrate using HPLC

In a 60-mm dish, 1 M L-sodium lactate solution (8 ml) was treated with plasma (L = 3 mm, 2.0 slm) for 15 min. The PAL was purified by HPLC on an Intrada Amino Acid Column (250 mm × 3 mm, Imtakt Corporation, Kyoto, Japan) at a flow rate of 0.8 ml/min with ultraviolet detection as previously reported^31^ (Fig. 4a). For 2,3-dimethyltartrate purification, a gradient was prepared with solvent A (acetonitrile containing 0.3% formic acid) and solvent B (H_2_O containing 100 mM ammonium formate) as follows: the gradient started at 0% B at 0 min, increased to 100% B after 0–30 min, remained at 100% B after 30–35 min, then decreased to 0% B after 35–45 min. Fractions containing the purified 2,3-dimethyltartrate (14-to-15-min fractions, Fig. 4a, S2) were lyophilized after neutralization, and subjected to the mass spectrometric analysis after dissolution in 1 ml of water. The mass spectrometric analysis was performed using a TQD Xevo triple-stage quadrupole mass spectrometer (Waters, Milford, MA) equipped with an ACQUITY ultra-performance liquid chromatography system (Waters, Fig. 3a) as previously described^32^. The samples (injection volume of 10 μl each) were separated on Intrada Amino Acid Columns (100 mm × 2 mm, Imtakt Corporation) at a flow rate of 0.3 ml/min. A gradient was prepared using mobile phases A (acetonitrile containing 0.1% formic acid) and B (100 mM ammonium formate) as follows: the gradient started at 1% B from 0 to 2 min, increased to 99% B after 2–12 min, remained at 99% B after 12–13 min, then decreased to 1% B after 13–15 min. Mass spectrometric analysis in the negative ion mode was performed with the selected ion recording mode (cone potential: 25 eV). The monitored selected ion recording transitions were as follows: L-sodium lactate, *m/z* 89; and 2,3-dimethyltartrate, *m/z* 177.

### Mitochondrial membrane potential assay

100,000 U251 cells were seeded in a 12-well plate. On the following day, cells were treated with 5, 10, or 20 mM of synthesized 2,3-dimethyltartrate in DMEM for 6 h. Medium containing the synthesized 2,3-dimethyltartrate was replaced for 2 mM of JC-1 (DOJINDO LABORATORIES, Kumamoto, Japan), and incubated for 1 h at 37 °C in 5% CO_2_. Cells were then harvested and analyzed by flow cytometry (BD FACSCanto II, BD Biosciences, NJ, USA) to determine the relative fluorescence intensities of red/green.

### Statistical analysis

All data are presented as the mean ± standard deviation. Statistical analysis of differences between groups was performed using the Student’s *t* test. A *P* value < 0.05 was considered to indicate a significant difference.

## Supplementary Information

Supplementary Information.

## Data Availability

The data that support the findings of this study are available from the corresponding author upon reasonable request.

## References

[1] Keidar M (2013). Cold atmospheric plasma in cancer therapy. Phys. Plasmas.

[2] Laroussi M (2009). Low-temperature plasmas for medicine?. IEEE Trans. Plasma Sci..

[3] Fridman G (2008). Applied plasma medicine. Plasma Process. Polym..

[4] von Woedtke T, Metelmann HR, Weltmann KD (2014). Clinical plasma medicine: State and perspectives of in vivo application of cold atmospheric plasma. Contrib. Plasm Phys..

[5] Kong MG (2009). Plasma medicine: An introductory review. New J. Phys..

[6] Tanaka H (2018). Molecular mechanisms of non-thermal plasma-induced effects in cancer cells. Biol. Chem..

[7] Tanaka H (2018). New hopes for plasma-based cancer treatment. Plasma.

[8] Tanaka H (2017). State of the art in medical applications using non-thermal atmospheric pressure plasma. Rev. Mod. Plasma Phys..

[9] Kaushik NK (2018). Biological and medical applications of plasma-activated media, water and solutions. Biol. Chem..

[10] Tanaka H (2013). Plasma-activated medium selectively kills glioblastoma brain tumor cells by down-regulating a survival signaling molecule, AKT kinase. Plasma Med..

[11] Utsumi F (2013). Effect of indirect nonequilibrium atmospheric pressure plasma on anti-proliferative activity against chronic chemo-resistant ovarian cancer cells in vitro and in vivo. PLoS ONE.

[12] Adachi T (2014). Plasma-activated medium induces A549 cell injury via a spiral apoptotic cascade involving the mitochondrial-nuclear network. Free Radic. Biol. Med..

[13] Torii K (2014). Effectiveness of plasma treatment on gastric cancer cells. Gastric Cancer Off. J. Int. Gastric Cancer Assoc. Jpn. Gastric Cancer Assoc..

[14] Hattori N (2015). Effectiveness of plasma treatment on pancreatic cancer cells. Int. J. Oncol..

[15] Takeda S (2017). Intraperitoneal administration of plasma-activated medium: Proposal of a novel treatment option for peritoneal metastasis from gastric cancer. Ann. Surg. Oncol..

[16] Nakamura K (2017). Novel intraperitoneal treatment with non-thermal plasma-activated medium inhibits metastatic potential of ovarian cancer cells. Sci. Rep..

[17] Tanaka H (2016). Non-thermal atmospheric pressure plasma activates lactate in Ringer's solution for anti-tumor effects. Sci. Rep..

[18] Sato Y (2018). Effect of plasma-activated lactated ringer’s solution on pancreatic cancer cells in vitro and in vivo. Ann. Surg. Oncol..

[19] Tanaka H (2019). Oxidative stress-dependent and -independent death of glioblastoma cells induced by non-thermal plasma-exposed solutions. Sci. Rep..

[20] Nakamura K (2021). Preclinical verification of the efficacy and safety of aqueous plasma for ovarian cancer therapy. Cancers.

[21] Jiang L (2021). Lysosomal nitric oxide determines transition from autophagy to ferroptosis after exposure to plasma-activated Ringer’s lactate. Redox Biol..

[22] Bruggeman PJ (2016). Plasma–liquid interactions: A review and roadmap. Plasma Sources Sci. Technol..

[23] von Woedtke T, Metelmann HR (2015). Focus issue: “Plasma–liquid interactions: Key role in plasma medical research and new fields of application”. Clin. Plasma Med..

[24] Jablonowski H, Schmidt-Bleker A, Weltmann KD, von Woedtke T, Wende K (2018). Non-touching plasma-liquid interaction—Where is aqueous nitric oxide generated?. Phys. Chem. Chem. Phys..

[25] Takeda K, Ishikawa K, Tanaka H, Sekine M, Hori M (2017). Spatial distributions of O, N, NO, OH and vacuum ultraviolet light along gas flow direction in an AC-excited atmospheric pressure Ar plasma jet generated in open air. J. Phys. D Appl. Phys..

[26] Kurake N (2016). Cell survival of glioblastoma grown in medium containing hydrogen peroxide and/or nitrite, or in plasma-activated medium. Arch. Biochem. Biophys..

[27] Takahashi Y (2018). Reduced HeLa cell viability in methionine-containing cell culture medium irradiated with microwave-excited atmospheric-pressure plasma. Plasma Process. Polym..

[28] Liu Y (2020). Hydrogen peroxide in lactate solutions irradiated by non-equilibrium atmospheric pressure plasma. Plasma Sources Sci. Technol..

[29] Kunze M, Hartig A (2013). Permeability of the peroxisomal membrane: Lessons from the glyoxylate cycle. Front. Physiol..

[30] Natsume A (2005). IFN-beta down-regulates the expression of DNA repair gene MGMT and sensitizes resistant glioma cells to temozolomide. Can. Res..

[31] Sasatsuki H, Nakazaki A, Uchida K, Shibata T (2020). Quantitative analysis of oxidized vitamin B1 metabolites generated by hypochlorous acid. Free Radic. Biol. Med..

[32] Yoshitake J, Shibata T, Shimayama C, Uchida K (2019). 2-Alkenal modification of hemoglobin: Identification of a novel hemoglobin-specific alkanoic acid-histidine adduct. Redox Biol..

