# Supplementary material for: Low temperature plasma irradiation products of sodium lactate solution that induce cell death on U251SP glioblastoma cells were identified
Source: Sci Rep. 2021 Sep 16;11:18488. doi: 10.1038/s41598-021-98020-w (PMC8446009; doi:10.1038/s41598-021-98020-w)
Supplement: Supplementary file 1 — Supplementary Information. [file 41598_2021_98020_MOESM1_ESM.docx]

**Low temperature plasma irradiation products of sodium lactate solution that induce cell death on U251SP glioblastoma cells were identified.**

**Hiromasa Tanaka^1, *^, Yugo Hosoi^2^, Kenji Ishikawa^1^, Jun Yoshitake^3^, Takahiro Shibata^3, 4^, Koji Uchida^3, 4, 5^, Hiroshi Hashizume^6^, Masaaki Mizuno^7^, Yasumasa Okazaki^8^, Shinya Toyokuni^1, 8^, Kae Nakamura^1, 9^, Hiroaki Kajiyama^1, 9^, Fumitaka Kikkawa^1, 9^, and Masaru Hori^1^**

^1^ Center for Low-temperature Plasma Sciences, Nagoya University, Furo-cho, Chikusa-ku, Nagoya 464-8601, Japan

^2^ Graduate School of Engineering, Nagoya University, Furo-cho, Chikusa-ku, Nagoya 464-8603, Japan

^3^ Institute of Nano-Life-Systems, Institute of Innovation for Future Society, Nagoya University, Nagoya 464-8601, Japan

^4^ Graduate School of Bioagricultural Sciences, Nagoya University, Furo-cho, Chikusa-ku, Nagoya 464-8603, Japan

^5^ Department of Applied Biological Chemistry, Graduate School of Agricultural and Life Sciences, The University of Tokyo, 1-1-1 Yayoi, Bunkyo-ku, Tokyo 113-8657, Japan

^6^ Institutes of Innovation for Future Society, Nagoya University, Furo-cho, Chikusa-ku, Nagoya 464-8601, Japan

^7^ Center for Advanced Medicine and Clinical Research, Nagoya University Hospital, 65 Tsurumai-cho, Showa-ku, Nagoya 466-8550, Japan

^8^ Department of Pathology and Biological Responses, Nagoya University Graduate School of Medicine, 65 Tsurumai-cho, Showa-ku, Nagoya 466-8550, Japan

^9^ Department of Obstetrics and Gynecology, Nagoya University Graduate School of Medicine, 65 Tsurumai-cho, Showa-ku, Nagoya 466-8550, Japan

*To whom correspondence should be addressed.

E-mail: [htanaka@plasma.engg.nagoya-u.ac.jp](mailto:htanaka@plasma.engg.nagoya-u.ac.jp)


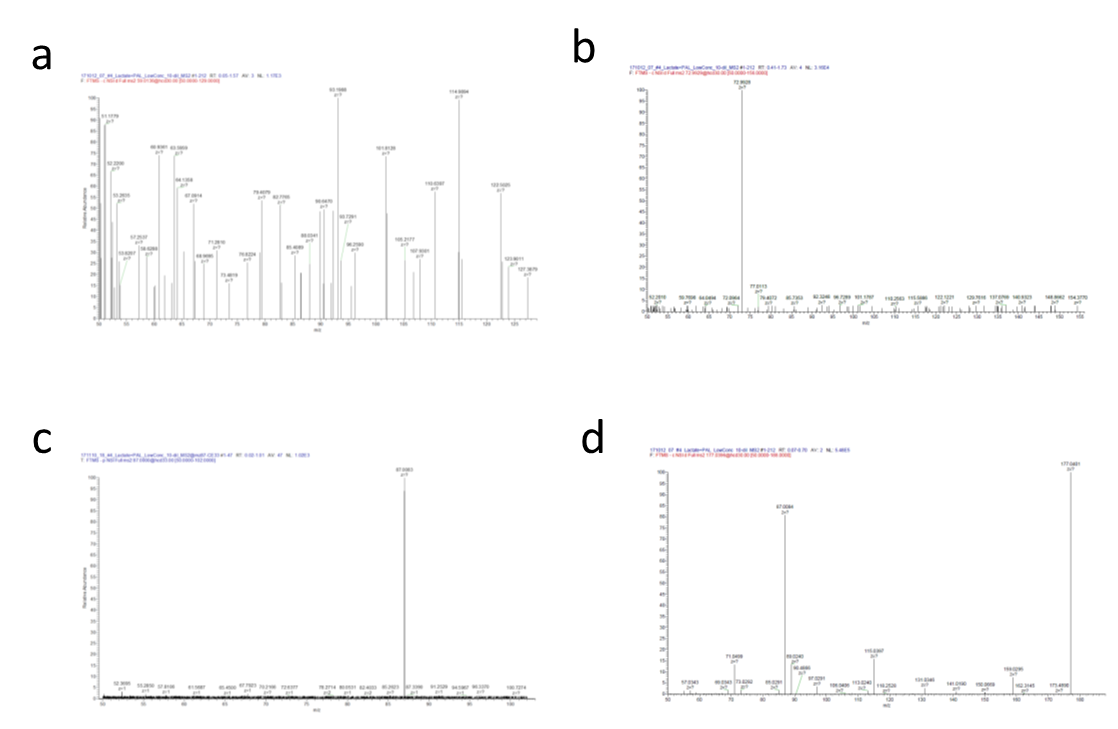


**Figure S1: MS/MS spectra detected in plasma-treated L-sodium lactate solution. (**a) Overall spectrum. (b) Spectrum derived from glyoxylic acid. (c) Spectrum derived from pyruvic acid. (d) Spectrum derived from 2,3-dimethyltartaric acid and pyruvic acid.


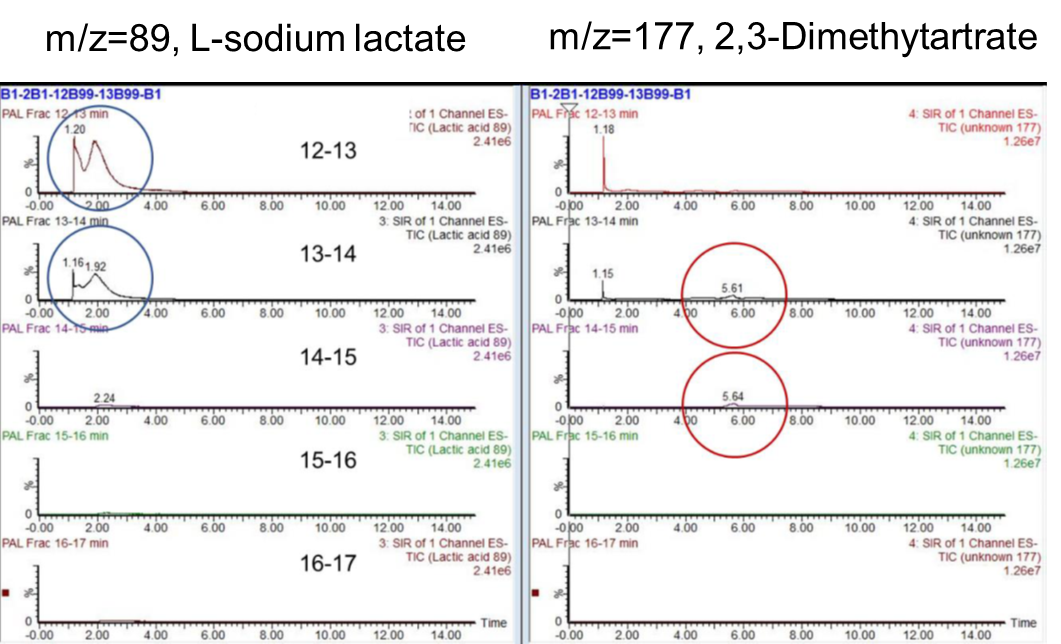


**Figure S2: MS/MS spectrum of each fraction.** L-sodium lactate was detected in the 12-to-13-min and 13-to-14-min fractions. 2,3-Dimethyltartrate was detected in the 13-to-14-min and 14-to-15-min fractions.
